# Using Historical and Experimental Data to Reveal Warming Effects on Ant Assemblages

**DOI:** 10.1371/journal.pone.0088029

**Published:** 2014-02-04

**Authors:** Julian Resasco, Shannon L. Pelini, Katharine L. Stuble, Nathan J. Sanders, Robert R. Dunn, Sarah E. Diamond, Aaron M. Ellison, Nicholas J. Gotelli, Douglas J. Levey

**Affiliations:** 1 Department of Biology, University of Florida, Gainesville, Florida, United States of America; 2 Department of Biological Sciences, Bowling Green State University, Bowling Green, Ohio, United States of America; 3 Department of Ecology and Evolutionary Biology, University of Tennessee, Knoxville, Tennessee, United States of America; 4 Department of Biological Sciences, North Carolina State University, Raleigh, North Carolina, United States of America; 5 Harvard Forest, Harvard University, Petersham, Massachusetts, United States of America; 6 Department of Biology, University of Vermont, Burlington, Vermont, United States of America; 7 National Science Foundation, Arlington, Virginia, United States of America; Centro de Investigación y de Estudios Avanzados, Mexico

## Abstract

Historical records of species are compared with current records to elucidate effects of recent climate change. However, confounding variables such as succession, land-use change, and species invasions make it difficult to demonstrate a causal link between changes in biota and changes in climate. Experiments that manipulate temperature can overcome this issue of attribution, but long-term impacts of warming are difficult to test directly. Here we combine historical and experimental data to explore effects of warming on ant assemblages in southeastern US. Observational data span a 35-year period (1976–2011), during which mean annual temperatures had an increasing trend. Mean summer temperatures in 2010–2011 were ∼2.7°C warmer than in 1976. Experimental data come from an ongoing study in the same region, for which temperatures have been increased ∼1.5–5.5°C above ambient from 2010 to 2012. Ant species richness and evenness decreased with warming under natural but not experimental warming. These discrepancies could have resulted from differences in timescales of warming, abiotic or biotic factors, or initial species pools. Species turnover tended to increase with temperature in observational and experimental datasets. At the species level, the observational and experimental datasets had four species in common, two of which exhibited consistent patterns between datasets. With natural and experimental warming, collections of the numerically dominant, thermophilic species, *Crematogaster lineolata,* increased roughly two-fold. *Myrmecina americana,* a relatively heat intolerant species, decreased with temperature in natural and experimental warming. In contrast, species in the *Solenopsis molesta* group did not show consistent responses to warming, and *Temenothorax pergandei* was rare across temperatures. Our results highlight the difficulty of interpreting community responses to warming based on historical records or experiments alone. Because some species showed consistent responses to warming based on thermal tolerances, understanding functional traits may prove useful in explaining responses of species to warming.

## Introduction

Global climatic change has altered phenology, ranges of individual species, and community structure across many taxa (reviewed in [1]). Predicting how species assemblages will change as a result of climatic warming is a prerequisite for understanding the ecological future, but such predictions remain vexingly difficult. Observational studies of relationships between climatic trends or weather events and changes in biotic assemblages have a long history in ecology [2]–[6]. When historical data exist, repeated sampling and comparisons of historical and contemporary datasets can reveal assemblage-level changes that have occurred concurrent with decades of climatic change [2], [7]–[11]. In essentially all cases, however, confounding factors (e.g., succession, pollution, changes in soil, invasion, landscape context) make it difficult to attribute observed differences solely to changes in climate [1].

Manipulative field experiments that simulate projected climatic change can provide a bridge between observational, correlative studies and potential mechanisms that underlie any observed patterns. These studies increase the ability to assign causation of biotic changes to abiotic variables. However, manipulative field experiments have their own limitations, such as limited replication and relatively small spatial and temporal scales. Experimental plots or chambers may not capture rare extremes in weather or interactions among climatic drivers [12]. Climatic changes in these experiments occur at a shorter time scale, so experiments may miss biotic changes that are slow to emerge. Likewise, high variation in intra- and interspecific responses may mask overall changes in community composition or diversity that may occur in the long term. A combined approach of long-term observations and experimental manipulations can overcome many of the inherent limitations of detection and attribution of each approach in isolation [13], [14]. A challenge of such combined approaches, however, is that they depend on a combination of data from long-term observations and from warming (or other global change) experiments in the same region on the same taxa.

Here we revisit a set of sites where ant assemblages were sampled ∼35 years ago and compare changes in these assemblages through time to results from an ongoing warming experiment on an assemblage of ants in the same region. We focus on ants because they are diverse, abundant, ecologically important [15], well studied in the southeastern United States [16], and because they are among the very few animal taxa to be studied in field manipulations of climatic change [17]. Further, temperature influences many aspects of ant biology, including assemblage-level metrics such as species diversity [18]–[20], and population-level phenomena such as the timing of reproduction [21], dynamics of foraging behavior [22]–[24], limits of species ranges [25]–[27], and colony growth and development [28]. Specifically, we asked whether the patterns observed from the long-term resampling of ant assemblages over 35 years are congruent with results from an ongoing warming experiment that has been running continuously since 2010.

## Materials and Methods

### Study Systems

We conducted this study at two sites approximately 450 km apart: Savannah River Site (SRS), a National Environmental Research Park, South Carolina, (33.21 N, 81.41 W; 80–130 m above sea level [29]) and Duke Forest, North Carolina (35.52 N, 79.59 W, 130 m above sea level). Permission to conduct this research was granted by the Office of the Duke Forest and the Savannah River Ecology Laboratory. This research did not involve endangered or protected species. At SRS, our sampling areas were in two stands of turkey oak (*Quercus laevis*) forest (a map and description of the study site are in [30]). At Duke Forest, our experimental site is located in an oak-hickory (*Quercus*-*Carya*) forest.

### Historic Data: Savannah River Site

Data on ants at SRS were collected by Van Pelt and Gentry [30] in the summer (date unspecified) of 1976 and by one of us (JR) in the summers of 2010 and 2011, using the same sampling areas and similar methodology. Van Pelt and Gentry [30] used 148 mL (diameter not specified) plastic-vial pitfall traps baited with either sugar (30 traps) or peanut butter (10 traps) solutions. They also used baited containers and collected by hand, but because sampling effort for, and ant abundances obtained from these techniques were not reported, we used only their pitfall-trap data (“Scrub Oak” in Table 2 in [30]). In July 2010 and 2011, we sampled with 55 mL, 28.6 mm inner-diameter plastic-vial pitfall traps baited either with sugar (34 and 28 traps in 2010 and 2011, respectively) or peanut butter solutions (7 and 10) inserted into the forest floor flush with the ground surface. We placed traps 15 m apart along transects, interspersed in the sites described in Van Pelt and Gentry [30]. We randomized the placement of trap types. In both collection periods (one each year), pitfall traps were left open for 24 hours. We sorted and identified ants to species, except for two taxonomically difficult groups (*Solenopsis molesta* group and *Aphaenogaster rudis* complex) in which individuals were combined. To compare the two datasets we used current synonyms for species, based on the taxonomic history provided in Bolton’s [31] updated catalog at http://www.antwiki.org/wiki/New_General_Catalogue.

**Figure 1:**
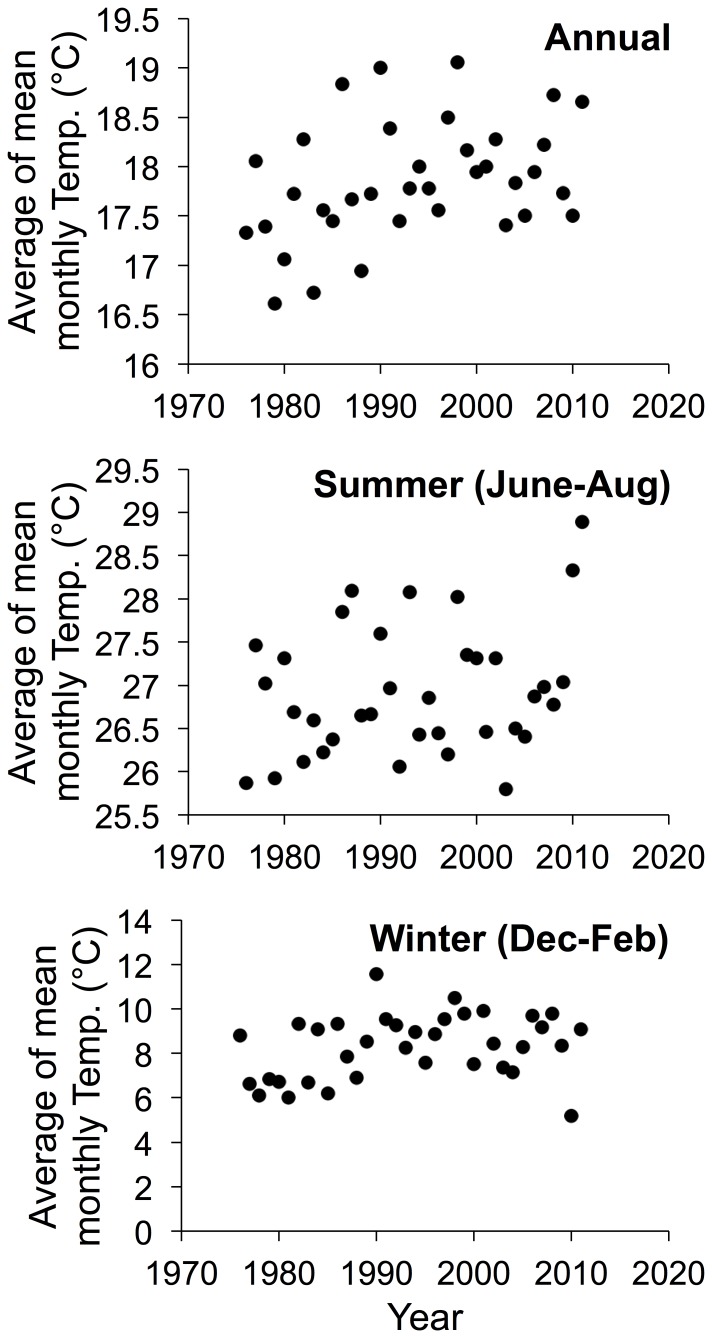
Annual, summer, and winter mean monthly temperatures near the Savannah River Site (South Carolina, United States) from 1976 to 2011.

To assess the extent of climatic warming between the historic and present-day sampling periods, we obtained data on monthly temperatures between 1976 and 2011 from the nearest weather station, Aiken 5SE, approximately 15 km away, in Aiken, SC (33.49N, 81.70W; SC State Climatology Office http://www.dnr.sc.gov/climate/sco/). Missing data (∼12% of months) were filled in using data from the second-nearest weather station, Bush Field (KAGS), approximately 25 km away, in Augusta, GA (33.38N 81.97W; National Oceanic and Atmospheric Administration; http://www.noaa.gov/). Mean summer temperatures (June, July, and August) were approximately 2.7°C warmer in 2010–2011 than in 1976 (Fig. 1). We recognize that extreme warm temperature anomalies, like those in southeastern United States in 2010 and 2011 are an important aspect of climate change [32]. Over the intervening years, mean annual temperatures also showed an increasing trend (Fig. 1).

### Duke Forest Warming Experiment

The Duke Forest warming experiment consists of 12 octagonal, open-top chambers, each built around a >20 cm dbh oak (*Quercus*) tree. Chambers are constructed of wooden frame walls covered in greenhouse sheeting. Each chamber is 5 m in diameter, 1.5 m high (∼ 22 m^2^), and has a 3-cm gap along the bottom to allow ants to enter and exit. Nine chambers have been heated with warm air since January 2010 in a regression design at 0.5°C intervals ranging from 1.5 to 5.5°C above ambient temperature; three additional chambers serve as ambient controls. Air temperature is monitored within each chamber. Details of the experimental design are provided in Pelini et al. [17]. Within each chamber, we have collected data on ants since 2009 using four pitfall traps (90 mL, 50 mm diameter) filled to approximately one-third of their volume with propylene glycol. Traps were left open for a 48-h sampling period. We identified collected ants to species and deposited voucher specimens at North Carolina State University. To correspond with SRS data, Duke Forest pitfall data from only summer months (June, July, and August) of 2010–2012 were used. Most of the ants detected in pitfall traps in the chambers come from colonies that are located in the chambers [33].

### Statistical Analyses

Data from pitfall traps provide a combined estimate of ant activity and density because a change in either activity or density will affect the rate at which ants fall into the traps [34]. Despite this drawback, pitfall traps are commonly used in studies such as ours because they are easy to standardize and have little impact on soil, litter, or ant populations [35], [36]. Because high numbers of individuals recruited to baited pitfall traps at SRS, we measured activity-density as number of traps with a given species present. In contrast, non-baited pitfall traps at Duke Forest did not recruit high numbers of workers and there were relatively few per chamber, so we measured activity-density as number of individuals per chamber of a given species. Relative activity-density (analogous to relative abundance) was calculated as the activity-density of a given species divided by the sum of the activity-densities of all species.

Differences in species richness between sampling periods at SRS were estimated from sample-based rarefaction (to adjust for sampling effort) on incidence data from both 2010 and 2011 pitfall trap data using EstimateS [37]. To estimate species evenness, we calculated Hurlbert’s PIE (probability of interspecific encounter, [38]). PIE varies between 0 and 1, with greater values indicating greater evenness. We used PIE as a metric of evenness because it is robust to differences in sample size and is intuitive to interpret as the probability that two individuals randomly drawn are from a different species. To estimate species turnover, we calculated Bray-Curtis distance (a measure of community dissimilarity) on relative activity-density of ant species [39] among sampling periods at SRS and among temperature treatments at Duke Forest. Bray-Curtis distance varies between 0 and 1, with greater values indicating greater dissimilarity between assemblages.

We examined the relationship between temperature and species richness, evenness, and the relative activity-density of each species that occurred at both sites. We also used Mantel tests with 10,000 permutations to examine the association between pairwise temperature differences and species turnover at both sites. For Duke Forest data, we used temperature differences among chambers. For SRS data, we used mean summer temperature differences among sampled years.

## Results

A total of 56 ant species was recorded across both periods and sites (Table 1; Table S1). Seventy-six percent of the species that occurred at Duke Forest were present at SRS, either in the samples collected for this study or other studies ([40], Resasco and Booher unpublished data). However, only four of these species occurred in historical samples, present-day samples at SRS, and present-day samples in the warming experiment: *Crematogaster lineolata, Myrmecina americana, Solenopsis molesta* (species group), and *Temnothorax pergandei*.

**Table 1:** Species list for Savannah River Site and Duke Forest for this study.

Species	SRS 1976	SRS 2010	SRS 2010	Duke Forest 2010–12
*Aphaenogaster ashmeadi* (Emery)	0.029	0.054	0.122	
*Aphaenogaster fulva* (Roger)				0.001
*Aphaenogaster lamellidens* (Mayr)				0.046
*Aphaenogaster mariae* (Forel)				0.001
*Aphaenogaster rudis* complex		0.172	0.130	0.301
*Aphaenogaster tennesseensis* (Mayr)				0.004
*Aphaenogaster treatae* (Forel)	0.089	0.022	0.008	
*Camponotus americanus* (Mayr)				0.006
*Camponotus castaneus* (Latreille)				0.034
*Camponotus chromaiodes* (Bolton)				0.024
*Camponotus nearcticus* (Emery)				0.002
*Camponotus pennsylvanicus* (DeGeer)			0.008	0.101
*Camponotus socius* (Roger)	0.077			
*Crematogaster ashmeadi* (Mayr)				0.008
*Crematogaster lineolata* (Say)	0.147	0.355	0.305	0.235
*Crematogaster minutissima* (Mayr)		0.000	0.008	
*Crematogaster vermiculata* (Emery)				0.002
*Dorymyrmex* sp.	0.019			
*Forelius pruinosus* (Roger)	0.040			
*Formica dolosa* (Buren)	0.010	0.054	0.023	
*Formica pallidefulva* (Latreille)		0.011	0.000	0.017
*Formica sanguinea* group				0.020
*Formica subsericea* (Say)				0.019
*Hypoponera opacior* (Forel)		0.011		
*Lasius interjectus* (Mayr)				0.001
*Myrmecina americana* (Weber)	0.039		0.008	0.050
*Myrmecina* sp.				0.002
*Neivamyrmex texanus* (Watkins)				0.027
*Nylandaria faisonensis* (Forel)		0.183	0.183	0.001
*Nylanderia arenivaga* (Wheeler)	0.019			
*Nylanderia parvula* (Mayr)	0.132	0.011		
*Nylanderia concinna* (Trager)				0.001
*Nylanderia terricola* (Buckley)				0.001
*Pheidole davisi* (Wheeler)	0.010			
*Pheidole dentata* (Mayr)	0.069	0.022	0.069	
*Pheidole dentigula* (Smith)		0.000	0.031	
*Pheidole metallescens* (Emery)	0.010			
*Pheidole morrisi* (Forel)	0.029	0.022		
*Pheidole crassicornis* (Emery)	0.050			
*Pogonomyrmex badius* (Latreille)	0.010			
*Ponera pennsylvanica* (Buckley)				0.018
*Prenolepis imparis* (Say)				0.005
*Pseudomyrmex ejectus* (Smith)	0.010			
*Stigmatomma pallipes* (Haldeman)				0.003
*Strumigenys bunki* (Brown)				
*Strumigenys carolinensis* (Brown)				0.001
*Strumigenys ornata* (Mayr)		0.022	0.008	0.001
*Strumigenys pergandei* (Emery)				0.001
*Strumigenys* sp (DFmorphX)				0.001
*Solenopsis molesta* group.	0.156	0.022	0.084	0.059
*Stenamma* cf. *impar*				0.001
*Stenamma impar* (Forel)				0.001
*Temnothorax pergandei* (Emery)	0.030	0.043	0.015	0.002
*Temnothorax schaumii* (Roger)				0.001
*Temnothorax curvispinosus* (Mayr)				0.013
*Trachymyrmex septentrionalis* (McCook)	0.010			

Values indicate species relative activity-density at the indicated site or time.

**Figure 2:**
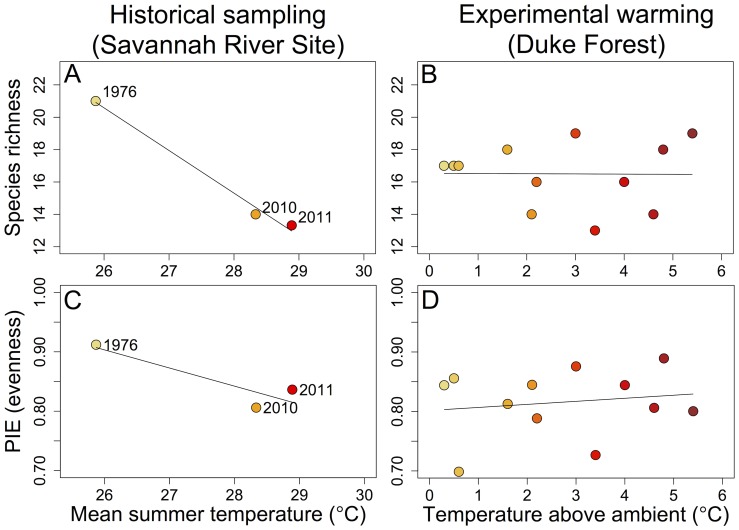
Relationships between temperature and ant diversity. Components of diversity are: species richness (A–B), evenness (C–D) at Savannah River Site (A, C) and Duke Forest (B, D). Dots for Savannah River Site represent sampling periods and dots for Duke Forest represent warming chambers. Warmer colors indicate warmer temperatures.

Estimated species richness decreased by approximately 35% at SRS between 1976 and 2010–2011 (33% by 2010; 37% by 2011). This difference is outside the present-day 95% confidence interval constructed after rarifying to equivalent sample sizes (1976 observed species richness: 21; 2010 and 2011 rarefied species richness 95% CI: 12–14; Fig. 2A). In contrast, three years of experimental warming at Duke Forest have, as of yet, shown no effect on species richness (ß = −0.01; SE = 0.35; r^2^<0.001; *P* = 0.99; Fig. 2B).

Evenness decreased by approximately 10% at SRS between 1976 and 2010–2011 (12% by 2010; 8% by 2011). This difference is outside of the present-day 95% confidence interval constructed after rarifying to equivalent sample sizes (1976 observed PIE: 0.91; 2010 and 2011 rarefied species richness 95% CI: 0.79–0.84; Fig. 2C). As with species richness, differences in evenness were not apparent in the experimental warming treatments at Duke Forest (ß = 0.005; SE = 0.01; r^2^ = 0.03; *P* = 0.62; Fig. 2D).

Bray-Curtis distance (species turnover) was positively related to mean summer temperature differences at SRS, although the relationship was not statistically significant (Mantel r = 0.90; *P* = 0.33). At Duke Forest we found that as temperature differences among warming chambers increased, Bray-Curtis distance tended to increase (Mantel r = 0.22; *P* = 0.06).

**Figure 3:**
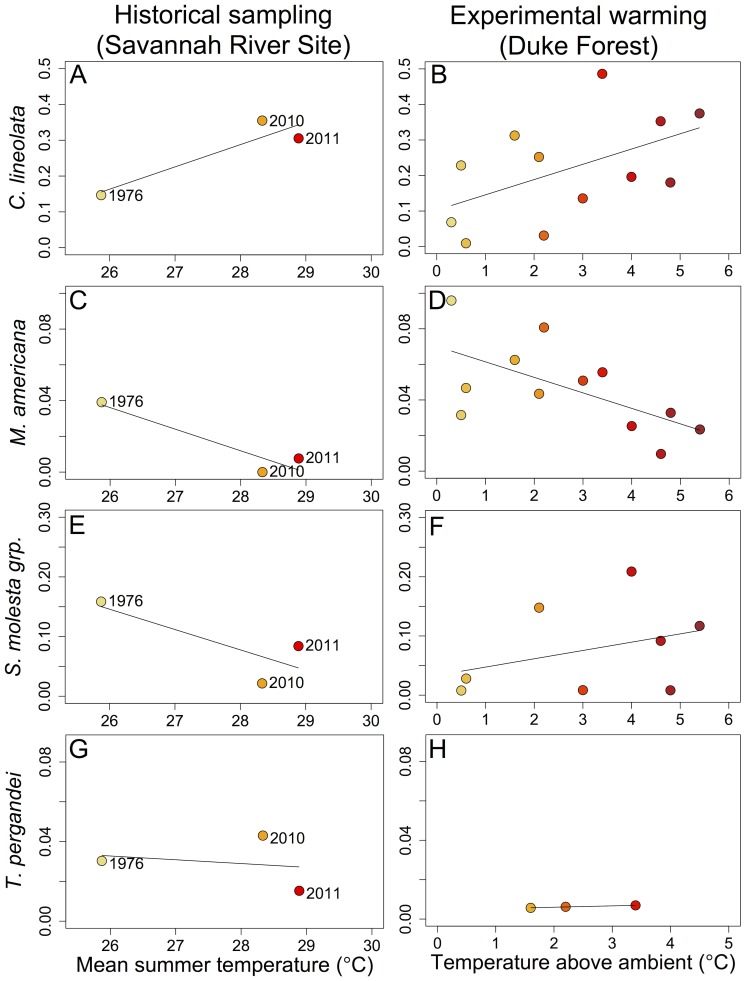
Relationships between temperature and species relative activity-densities for ant species that occurred at both Savannah River Site and Duke Forest. Species are: A–B) *Crematogaster lineolata*, C–D) *Myrmecina americana*, E–F) *Solenopsis molesta* group, G–H) *Temnothorax pergandei*. Dots for Savannah River Site represent sampling periods and dots for Duke Forest represent warming chambers where the species occurred. Warmer colors indicate warmer temperatures.

The relative activity-density of *Crematogaster lineolata* more than doubled between the 1976 sampling period and the present-day sampling period at SRS (Fig. 3A). Similarly, there was a trend towards a positive relationship between the extent of warming and *Crematogaster lineolata* relative activity-density in the experimental chambers at Duke Forest (ß = 0.04; SE = 0.02; r^2^ = 0.27; *P* = 0.08; Fig. 3B).

The relative activity-density of *Myrmecina americana* and the *Solenopsis molesta* species group decreased between sampling periods at SRS (Fig. 3C,E). At Duke Forest, the relative activity-density of *Myrmecina americana* also decreased (ß = −0.01; SE = 0.003; r^2^ = 0.39; *P* = 0.03; Fig. 3 D) but the relative activity-density of the *Solenopsis molesta* group did not show any relationship with temperature (ß = 0.01; SE = 0.015; r^2^ = 0.12; *P* = 0.40; Fig. 3F). *Temnothorax pergandei* was rare at both sites and did not appear to respond to warming at either site (Fig. 3G,H).

## Discussion

Our results, taken together, suggest that at least some of the long-term responses of species are congruent with the short-term responses of species to warming. Species turnover (Bray-Curtis distance) exhibited similar, positive trends at SRS and Duke Forest, although the increases were not statistically significant. When we looked at species-specific responses for four species collected in all sampling periods at both sites, we found similarities between SRS and Duke for the numerically dominant species, *Crematogaster lineolata* and for *Myrmecina americana* (Fig. 3A–D). Although we detected declines in ant species richness and evenness over a period of several decades at SRS, we did not find congruent results over a period of several years at the experimental warming site (Duke Forest; Fig. 2). Further, species in the *Solenopsis molesta* group responded variably across the sites, decreasing with time at SRS while not responding to temperature at Duke Forest. We note, however, that the poor taxonomy of the *Solenopsis molesta* species group complicates such comparisons.

Many factors may have contributed to the observed differences in the responses to warming of the ant assemblages at SRS and Duke Forest. The nature and timescale of warming is an obvious difference: at Duke Forest the warming was applied rapidly and maintained over a period of several years, whereas at SRS the warming was incremental and variable over 35 years. Differences in microclimate, local community structure, and land-use also may have played a role in driving variation in the responses to warming of the two assemblages. For example, there is evidence of successional maturation of forest stands at SRS that has not occurred during the three years of warming at Duke Forest. Van Pelt and Gentry [30] describe the habitat as a “subclimax forest” but do not provide vegetation data to allow a direct comparison with current conditions. Further, the presence of ant species such as *Dorymyrmex* sp., *Forelius pruinosus, Nylanderia arenivaga, Pheidole davisi, Pheidole metallescens, Pheidole crassicornis, Pogonomyrmex badius,* and *Trachymyrmex septentrionalis* in the 1976 SRS data but not the present-day data suggests that the sampling sites likely were more open and xeric during the original sampling period. This potential difference is important because succession can result in large changes in animal assemblages [41], [42] including assemblages of ants [43].

Other potential drivers include altered precipitation regimes, changes in leaf litter, and shifts in species interactions. The important point is that observed shifts in the ant assemblage at SRS have multiple explanations, whereas those at Duke Forest most parsimoniously are attributable directly to warming. We also have reported high intra- and interspecies variation in ant responses to warming in the Duke Forest warming experiment [24], [25], [33], [44]–[46]. Such high variation in short-term responses of ants to warming may mask patterns of diversity and composition that eventually result in the longer term.

For the subset of species shared among the SRS and Duke Forest samples, physiological tolerance appears to explain some of their responses to warming. The activity-density of the numerically dominant species, *Crematogaster lineolata*, increased with warming at both SRS and Duke Forest (Fig. 3A,B). In addition, samples at Duke Forest have documented greater nest box colonization by this species in the warmest chambers (unpublished data). Previous work from the Duke Forest site and surrounding areas has linked increasing abundance and foraging intensity of *C. lineolata* with greater tolerance of high temperatures than that of co-occurring species [24], [25], [47]. Indeed, *C. lineolata* has one of the highest critical thermal maxima (temperature of loss of ant muscular coordination), 46.1°C, among the 20 most common species at Duke Forest (data at: http://harvardforest.fas.harvard.edu:8080/exist/xquery/data.xq?id=hf113). In contrast, *Myrmecina americana* showed congruent declines with temperature at SRS and at Duke Forest (Fig. 3 C,D). This species has the lowest critical thermal maximum, 38.8°C, among the species collected at Duke Forest.

The increased activity-density of *Crematogaster lineolata* in both the observational and the experimental studies might lead to subsequent effects on the rest of the ant assemblage. For instance, if *Crematogaster lineolata* benefits from warming by increasing foraging, evenness of the assemblage could be reduced by competitive displacement [44]; such results have also been observed for plant assemblages [48]. Effects of warming on species interactions have been demonstrated in a variety of aquatic and terrestrial systems [49], [50] including plant-herbivore [51], host-parasitoid [52], and trophic interactions [14], [53]–[56], ultimately influencing the composition of communities. Exploring how ongoing warming mediates interactions among species and in turn influences the structure and dynamics of species assemblages is a central challenge in global-change research [49], [50].

In summary, our results from observational data of shifts in activity-density in two individual ant species were similar to those of the experimental data, but overall responses of ant assemblages largely differed between observational and experimental studies. Species-specific responses may be linked to functional traits [57] such as thermal tolerance [24], [25], [57], whereas uncontrolled variables in observational studies and site-specific differences may mask assemblage-level changes [12]. Our study highlights challenges and the importance of assessing alternative explanations when drawing on experimental data to make stronger inferences from historical datasets about impacts of climate change.

## Supporting Information

Table S1 **Relative activity-densities of Duke Forest ant species among chamber treatments and years.**
(XLSX)
